# The Transactions of the American Medical Association, for 1852

**Published:** 1853-05

**Authors:** 


### EDITORIAL DEPARTMENT.

*The Transactions of the American Medical Association, for* 1852.—A copy of this work, which had not reached us at the time of our last issue, was duly forwarded to our address, but had not been delivered. It makes a volume of between nine and ten hundred pages. Its appearance has been greatly delayed owing to circumstances which the publishing committee could not control. The preface states that the greater portion of the causes retarding the publication might have been avoided had all the parties interested been as assiduous in securing its appearance as were the committee of publication.

The first seventy-two pages of the work are occupied by the minutes of the fifth annual meeting of the association.

The prize essay next follows, covering fifty-four pages.

Then follow Reports of Committees on Special Scientific Subjects. These reports consist of — 1. An essay "on the blending and conversion of types in fever." By Samuel Henry Dickson, M. D., of Charleston, S. C. This is an ingenious and interesting disquisition, but it may, perhaps, be questioned whether it will tend to settle doubtful questions of pathology pertaining to the subject of which it treats. 2. "On the action of water on lead pipes and the diseases proceeding from it." By Horatio Adams, M. D., of Waltham, Mass. We have read this report with much interest. The facts collected are of great practical consequence, and will serve to call the attention of the profession to the poisonous effects of lead, which are often not recognized, as such, in practice, and consequently persist in default of the appropriate management, and from the continued operation of the cause. We quote the last paragraph of the report: "In conclusion, your committee will only add that the settled conviction to which their labors, in preparing this report, have conducted them, is this: that it is never safe to use water drawn through lead pipes, or stored in the leaden cisterns for domestic purposes, and that any article of food or drink is dangerous to health which by any possibility can be impregnated with saturnine matter. It may possibly be done in some cases with impunity, but it is *impossible to predetermine* the cases of safety where so many are fraught with danger." 3. "On permanent cure of Reducible Hernia." By George Hayward, M. D., of Boston, Mass. This, also, is an interesting report. The subject is treated practically and ably, as would be expected from the character of the distinguished surgeon by whom it was prepared, as well as that of each of the gentlemen (Drs. J. Mason Warren and S. Parkman) associated on the committee. The following are the opinions at which the committee arrived after a careful examination of the subject, having obtained the views of several other eminent surgeons: "I. That there is no surgical operation at present known which can be relied on with confidence, to produce, in all instances, or even in a large proportion of cases, a radical cure of reducible hernia." "II. That they regard the operation of injection by the subcutaneous method as the safest and best. This will probably, in some cases, produce a permanent cure, and in many others will afford great relief." "III. That compression, when properly employed, is, in the present state of our knowledge, the most likely means of effecting a radical cure in the greatest number of cases." 4. "On Water; its topical uses in

Surgery." By Charles A. Pope, M. D., of St. Louis, Mo. We have not bestowed on this report, as yet, the careful attention which it claims. On a superficial examination it appears to us to be a timely contribution of judicious views on a topic of not a little practical interest.

The foregoing reports extend over one hundred and fifty-eight pages. They will be read, we doubt not, with satisfaction, and generally considered valuable contributions to our medical literature. For ourselves, however, we must confess that we should have preferred the reports on the progress of the several departments of medical science, after the plan originally adopted by the association. The change from these reports, to those on special subjects, seems to us to have been injudicious. This was the view taken by us at its first announcement, and the examination of the present volume of Transactions has served to strengthen this opinion. We sincerely hope the association will go back to the old system, which we are convinced would be more acceptable and useful, than the present, to the great majority of medical readers.

More than four hundred pages of the work are devoted to reports on Epidemics in different States. Of the value of these reports we are not prepared to speak. That they possess some, and it may be considerable value is possible, but by most medical readers they will never be read. We doubt the policy of filling so much of the volume of the Transactions with merely historical facts of this description. They are important we concede, but what concerns the profession is, not the details, for which few have any taste, but the conclusions to be deduced therefrom. The practical reader naturally inquires, on looking over so large a collection of facts, "well, what does it all amount to; what shall I gain if I take the pains to read all this matter?" A candid consideration of this question will, we fear, seldom lead to the resolution to enter on the task of a perusal.

The last two hundred and more pages are occupied by a "Report on Medical Botany." By A. Clapp, M. D., chairman of committee. This will, undoubtedly, be useful for reference, and is evidently the result of much labor on the part of the author.

As a whole, we continue to look on the annual volume of Transactions with pleasure and pride, and we earnestly hope that the evidence which is thus afforded of the utility of the association, will, for a long time to come, be reproduced with each returning year.

